# Development and functional characterization of a novel respiratory mask with full accordion cushioning to prevent air leaks and pressure injuries during non-invasive ventilation

**DOI:** 10.1186/s13054-024-05133-5

**Published:** 2024-11-01

**Authors:** Masahiko Hara, Masatake Tamaki

**Affiliations:** 1 Department of Medical Device Development, iDevice, Inc., Osaka, Japan; 2 Center for Community-Based Healthcare Research and Education, Shimane University Faculty of Medicine, Izumo, Japan

## Dear editor,

**Fig. 1:**
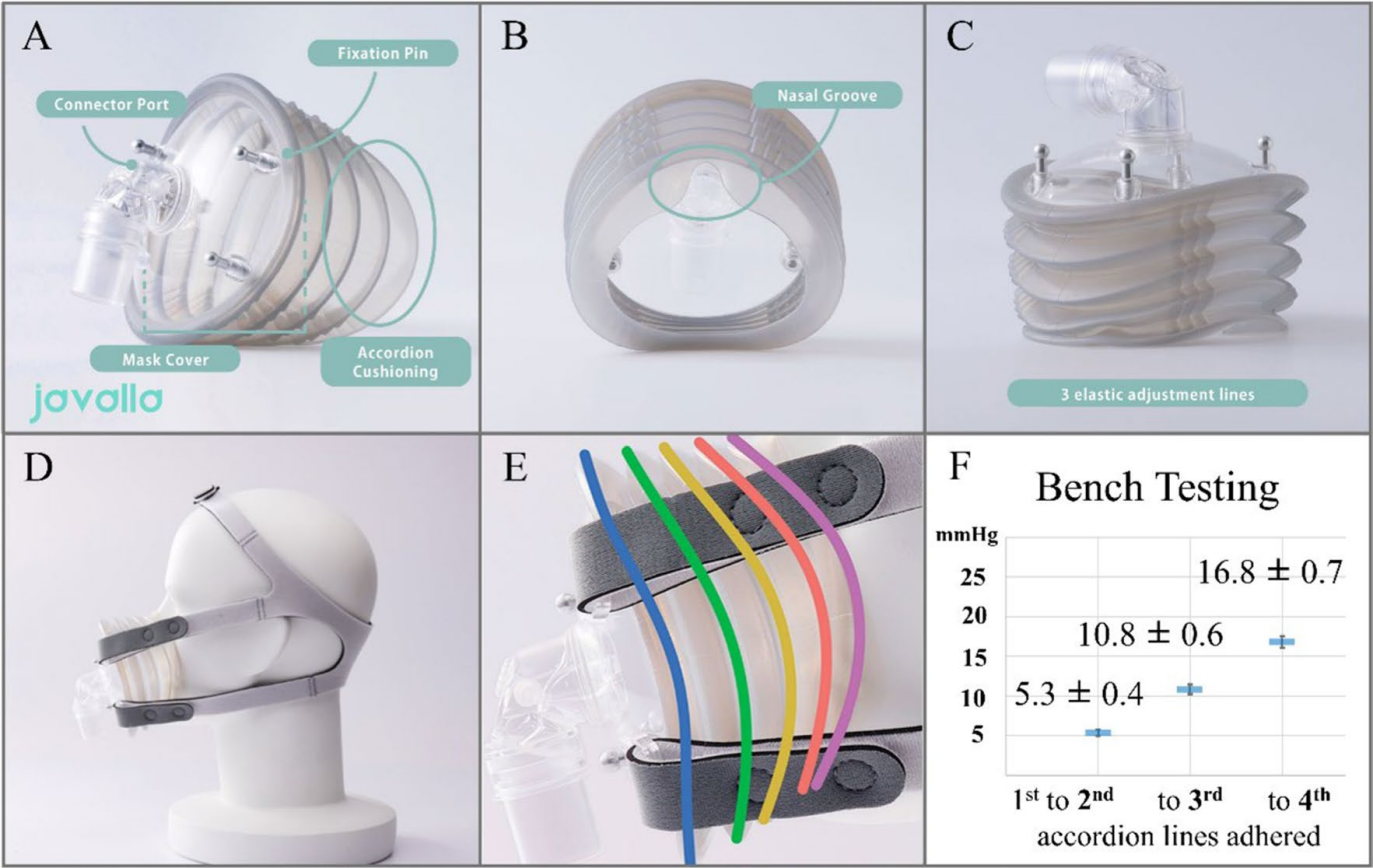
Structural and Functional Features of Our Novel Respiratory Mask with Full Accordion Cushioning. Overview of the mask (**A**). The rear view of the mask from the face side shows the nasal groove (**B**). Elastic adjustment lines are symmetrically aligned with six on the nasal side and eight on the chin side (**C**). An illustration of the mask in clinical use (**D**). A detailed view of the accordion structure, showing the five outermost ridges defined as accordion lines (**E**). These ridges are sequentially labeled from the face side (first line, purple) to the cover side (fifth line, blue). Mechanical testing provided visual cues for the estimated pressure at which the mask would adhere to the skin (**F**). See Video. S1 for the 3-dimensional computer-aided design data of the mask, Video. S2 for a frontal view of the mask in use, Video. S3 for a visualization of the nasal groove, and Video. S4 for the folding function

Non-invasive ventilation (NIV) is critical in the treatment of several respiratory diseases [1, 2]. However, interface air leakage and resultant pressure injury from tight-fitting can lead to intolerance or unsuccessful implementation of NIV [3, 4]. In response to these challenges, we have developed a new type of oronasal mask with full accordion cushioning designed to achieve effective sealing at lower pressures (Fig. 1 and Video. S1). Our mask incorporates six innovations: full accordion cushioning, turtle shell cover, nasal groove, folding function, visual pressure indicator, and soft medical-grade silicone (Videos. S2, S3, and S4). The mask is tapered toward the face, and it also incorporates multiple elastic adjustment lines to improve adaptability and fit, allowing the mask to “fold” snugly around the face. These elements enhance the mask’s ability to evenly distribute pressure and conform to different facial shapes, providing a secure fit at low pressures. The thickness of the accordion cushion decreases toward the face side, providing a visual indication of pressure application through the compression of the accordion valleys.

To assess the mask’s performance, a mechanical bench test was conducted to evaluate sealing efficiency and to estimate skin pressure at various visual pressure indicator scenarios. Smoke leak tests were performed to visually confirm the seal (Video. S5). The mask conformed effectively to various mannequin head shapes, and achieved complete sealing at an estimated skin pressure of 2.2 mmHg. The outermost ridges were sequentially labelled from the facial side. Estimated skin pressures were 5.3 ± 0.4 mmHg when the first and second accordion lines adhered, 10.8 ± 0.6 mmHg when the first through third lines adhered, and 16.8 ± 0.7 mmHg when the first through fourth lines adhered. The Lin’s concordance correlation coefficient was 0.987 (95% confidence interval, 0.963–0.995), indicating a high degree of agreement between measurements by different observers. Based on these results, we developed a special strap (Fig. S1) that allows the mask to be held in place on the face with minimal pressure, thereby improving usability. The mask was registered as a medical device in Japan under the product name “javalla” (iDevice, Inc., Osaka, Japan), a term that reflects the accordion-like structure. The mask is designed for reuse, with durability guaranteed for up to 10 patients per mask.

Initial clinical feedback has been overwhelmingly positive, highlighting the mask’s ease of fit and comfort without the need for specific sizing. The one-size-fits-all design eliminates the need for sizing by conforming to a variety of facial shapes, including different nasal contours. Users reported fewer air leaks, less discomfort, and reduced ventilator alarms. The folding feature benefited patients with edentulous faces or sunken cheeks. During the trial sales phase, 33 out of 74 hospitals (44.6%) adopted our product, despite its price being more than double that of the most commonly used masks available to them. However, some users noted a learning curve for the placement method, concerns about the looser fit, and the importance of keeping the ventilator tube tension-free (gravity-free) to prevent dislodgement due to the soft wearing of the mask. It was also observed that some users tended to secure the mask too tightly, as with conventional masks, resulting in excessive tightening that caused the 1st through 4th accordion lines to adhere. This led to the loss of the cushion’s flexibility, paradoxically increasing air leaks rather than preventing it.

This novel mask addresses critical challenges in NIV, such as air leak and pressure injury, which are often associated with high morbidity and increased healthcare costs [3, 4]. Pressure injuries occur when the sustained external force exceeds tissue perfusion pressure, typically around 30–35 mmHg [4, 5]. Our mask’s ability to provide an effective seal at low pressures shows potential in reducing the occurrence of these injuries. Preliminary testing suggests that the mask achieves an optimal balance between comfort and sealing efficacy when fitted to engage the first two to three accordion lines, maintaining pressures well below the injury threshold [4, 5]. The mask’s ease of fitting and elimination of sizing requirements could also reduce the time needed to implement NIV, particularly in emergency settings. As with any novel device, widespread clinical use and further studies are necessary to determine the long-term impact of the mask on clinical outcomes, including the prevention of pressure injuries and improving patient tolerance during NIV. In conclusion, we have developed a novel oronasal mask with full accordion cushioning to address air leakage, pressure discomfort, and pressure injury during NIV. Bench testing demonstrated effective sealing at lower pressures. Clinical studies are warranted to evaluate the impact of the mask on clinical outcomes.

## Supplementary Information

Additional file 1. Supplementary Figure 1. Demonstration of the Mask Fitting Process.

Additional file 2. Supplementary Video 1. Three-Dimensional Computer-Aided Design Visualization of Our Novel Respiratory Mask with Full Accordion Cushioning.

Additional file 3. Supplementary Video 2. How The Full Accordion Cushioning Conforms to The FACE.

Additional file 4. Supplementary Video 3. How The Nasal Groove Conforms to The FACE.

Additional file 5. Supplementary Video 4. The Folding Function in Action.

Additional file 6. Supplementary Video 5. Smoke-Based Air Leakage Assessment of The Mask.

## Data Availability

The data and materials used in this study are available from the corresponding author upon reasonable request.

## Abbreviation

NIV: Non-invasive ventilation
