# Supplementary material for: Development and functional characterization of a novel respiratory mask with full accordion cushioning to prevent air leaks and pressure injuries during non-invasive ventilation
Source: Crit Care. 2024 Nov 1;28:353. doi: 10.1186/s13054-024-05133-5 (PMC11531192; doi:10.1186/s13054-024-05133-5)
Supplement: Supplementary file 1 — Additional file 1. Supplementary Figure 1. Demonstration of the Mask Fitting Process. [file 13054_2024_5133_MOESM1_ESM.docx]

Supplementary Figure 1. Demonstration of the Mask Fitting Process


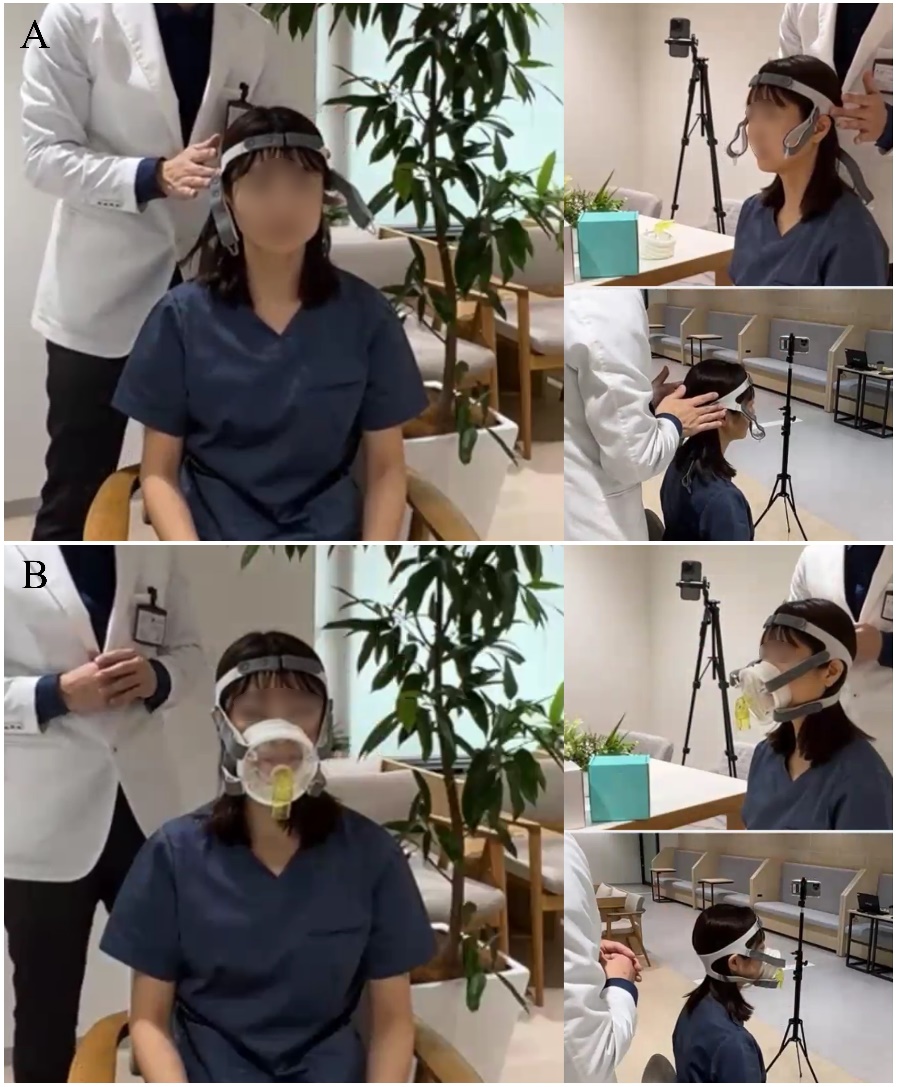


The mask is equipped with a special strap system designed to facilitate a new paradigm in mask fitting, in contrast to traditional tight-fitting methods. First, wide, soft elastic bands are secured to the forehead and occiput (A). This basic setup allows for the mask to be gently placed on the face without the need for forceful adjustments, creating a base for mask placement with minimal initial tension. After positioning the mask on the face, the top and bottom bands are attached to fixation pins and adjusted with only minimal tightening required during the initiation of non-invasive ventilation (B).

Supplementary Video 1. Three-Dimensional Computer-Aided Design Visualization of Our Novel Respiratory Mask with Full Accordion Cushioning


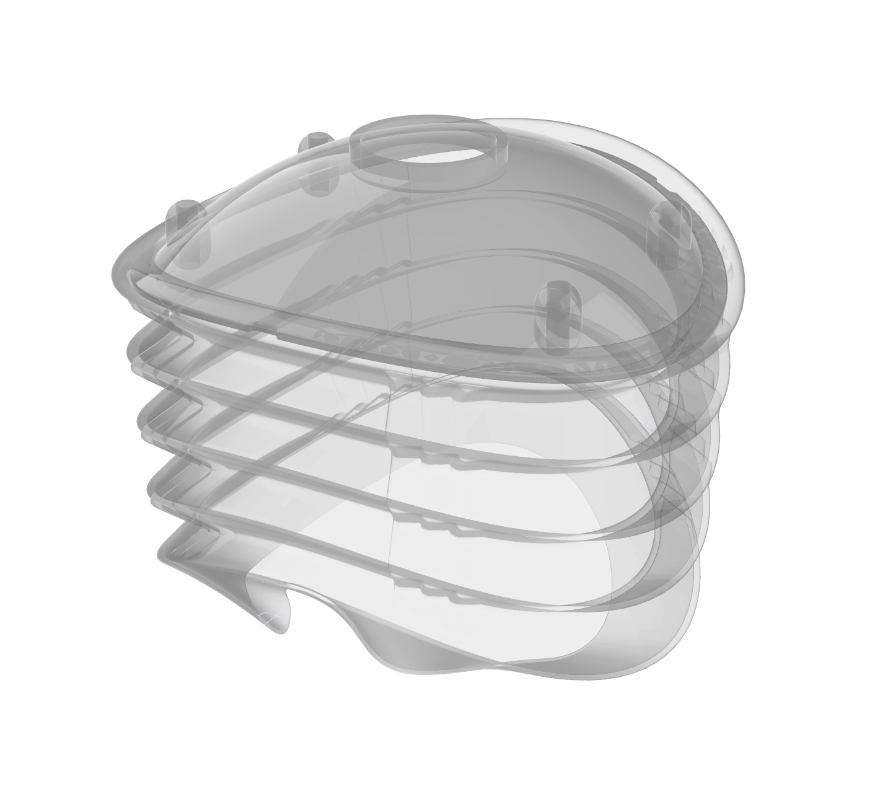


The video illustrates the structural features of the mask, including the full accordion cushioning, turtle shell cover, nasal groove, a slightly tapered mask body, and multiple elastic adjustment lines (eight on the chin side and six on the nasal side, symmetrically aligned).

<https://drive.google.com/file/d/1H93vvYqseneeFRaI532p4hfJRV3XMNhs/view?usp=sharing>

Supplementary Video 2. How The Full Accordion Cushioning Conforms to The FACE

This video, recorded at 6x slow motion, demonstrates how the silicone cushion gradually conforms and seals to the face, providing a secure fit with optimal pressure distribution.

<https://drive.google.com/file/d/1oInkyN5uT67NCIVeRxZf7aa4DJQn6m_k/view?usp=sharing>

Supplementary Video 3. How The Nasal Groove Conforms to The FACE

This video, recorded at 6x slow motion, demonstrates how the nasal groove gradually conforms and seals to the face, providing a secure fit with optimal pressure distribution.

<https://drive.google.com/file/d/1kE6tvJ1rgnC3JqyfrHynEwIBSC7TikZE/view?usp=sharing>

Supplementary Video 4. The Folding Function in Action

This video, recorded at 6x slow motion, demonstrates the cushion compresses around the nose and chin, molding to the face for a secure, wrap-around fit.

<https://drive.google.com/file/d/17uHXmWfLQ_jKwIr79hEEHEb1MEQud_lO/view?usp=sharing>

Supplementary Video 5. Smoke-Based Air Leakage Assessment of The Mask

This video demonstrates the air leakage test using smoke to evaluate the mask's sealing efficacy. Complete sealing was visually confirmed when the mask was gently placed on the mannequin’s face, indicating that the mask can achieve an effective seal at an estimated skin pressure of 2.2 mmHg. To estimate the pressure exerted on the skin by the mask, it was modeled as an ellipse with major and minor axes of 11.0 cm and 10.2 cm, respectively, resulting in a total area of 88.1 cm². The cushion thickness was assumed to be 1.7 cm along the horizontal axis and 2.2 cm along the vertical axis. The skin contact area was calculated by subtracting the area of an inner ellipse with dimensions of 9.3 cm on the major axis and 8.0 cm on the minor axis, resulting in an area of 58.4 cm². Thus, the effective contact area was estimated to be 29.7 cm². Given the weight of the mask of 90.0 g, the downward force due to gravity was calculated to be 0.88 N (0.09 kg × 9.81 m/s²). The pressure exerted on the skin was then determined by dividing this force by the contact area (0.88 N / 0.0030 m²), resulting in an estimated pressure of 293 Pa, equivalent to approximately 2.2 mmHg. Although the complex structure of the full accordion cushioning with its taper and multiple elastic adjustments may affect the actual pressure distribution, these simplified assumptions provide a practical approximation of the pressure exerted on the skin.

<https://drive.google.com/file/d/15MFhtGvKF4Tk1BoqWeVIgRaDvsLM2L2f/view?usp=sharing>
